# Supplementary material for: A simulation-based method to inform serosurvey design for estimating the force of infection using existing blood samples
Source: PLoS Comput Biol. 2023 Nov 27;19(11):e1011666. doi: 10.1371/journal.pcbi.1011666 (PMC10727435; doi:10.1371/journal.pcbi.1011666)
Supplement: S1 Text — (PDF) [file pcbi.1011666.s001.pdf]

Supporting Information

A simulation-based method to inform serosurvey design to estimate the force of infection using existing blood samples

Anna Vicco, Clare P. McCormack, Belen Pedrique, John H. Amuasi, Anthony Afum-Adjei Awuah, Christian Obirikorang, Nicole S. Struck, Eva Loren, Jürgen May, Isabela Ribeiro, Gathsaurie Neelika Malavige, Christl A. Donnelly, Ilaria Dorigatti

Table of Contents

Figures.. 2
Tables.. 9
Two-step testing approach... 15

Figures

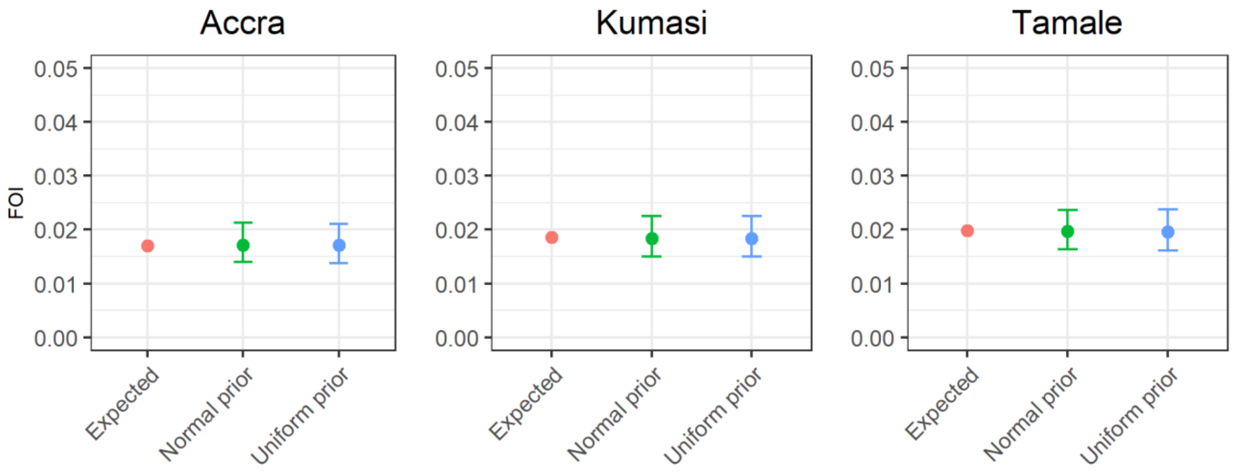

Figure A: Comparison of the FOI estimates obtained with the chosen scenario using different prior distributions. The plots show the median (point) and 95% CrI (error bar) of the FOI estimates obtained with the chosen scenario using model 1, respectively with a uniform prior distribution (blue) and a normal prior distribution (green) compared to the expected FOI from Cattarino et al. [3] (orange). The choice of the prior distribution does not influence the FOI estimate nor the width of the credible interval.

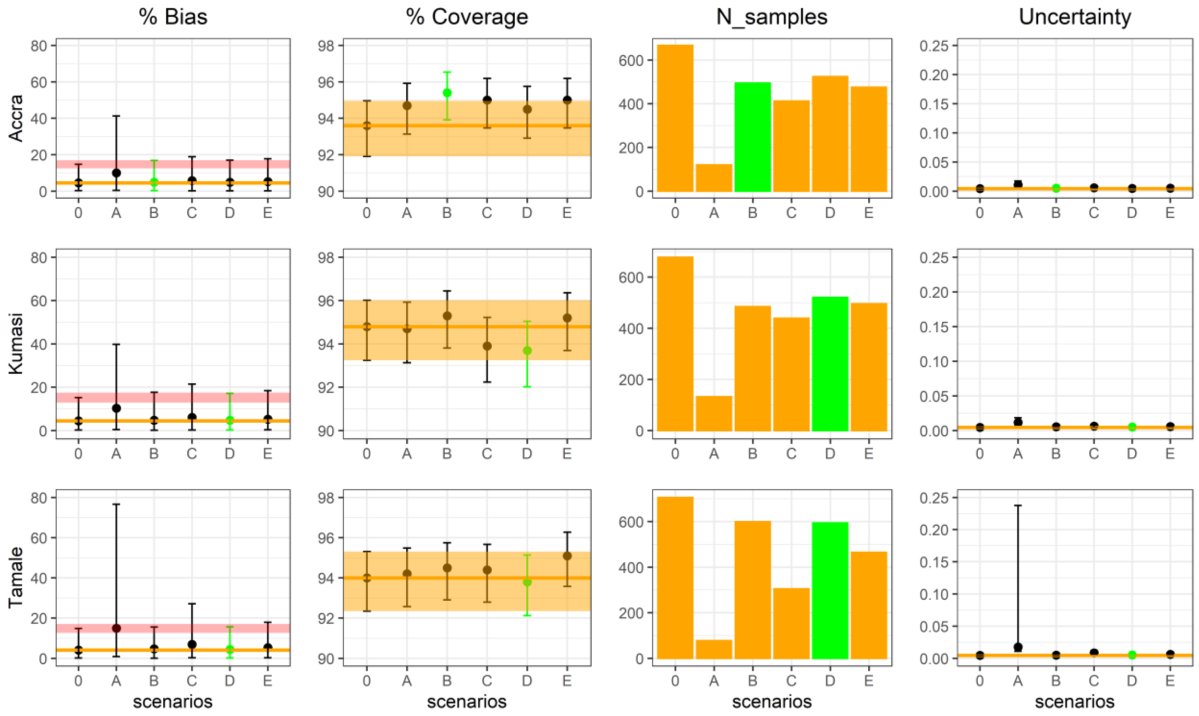

Figure B: Summary accuracy metrics obtained for Accra, Kumasi and Tamale with the 10-year age categories across scenarios. The four columns represent the bias, coverage, number of tested samples and uncertainty obtained for each scenario (x axis). The scenario highlighted in green indicates the selected scenario. The median bias and uncertainty (point) are reported with their 95% CrI (error bar) in columns 1 and 4, while the median coverage (point) is reported with its 95% exact binomial CI (error bar) in column 2. The horizontal orange line represents the median (columns 1, 2 and 4) and the orange ribbon represents the 95% CrI of the baseline scenario 0 (columns 2 and 4). The pink ribbon in the first columns represents the 15% tolerance around the upper bound of the 95% CrI of scenario 0, which was used in the first step of the selection criterion.

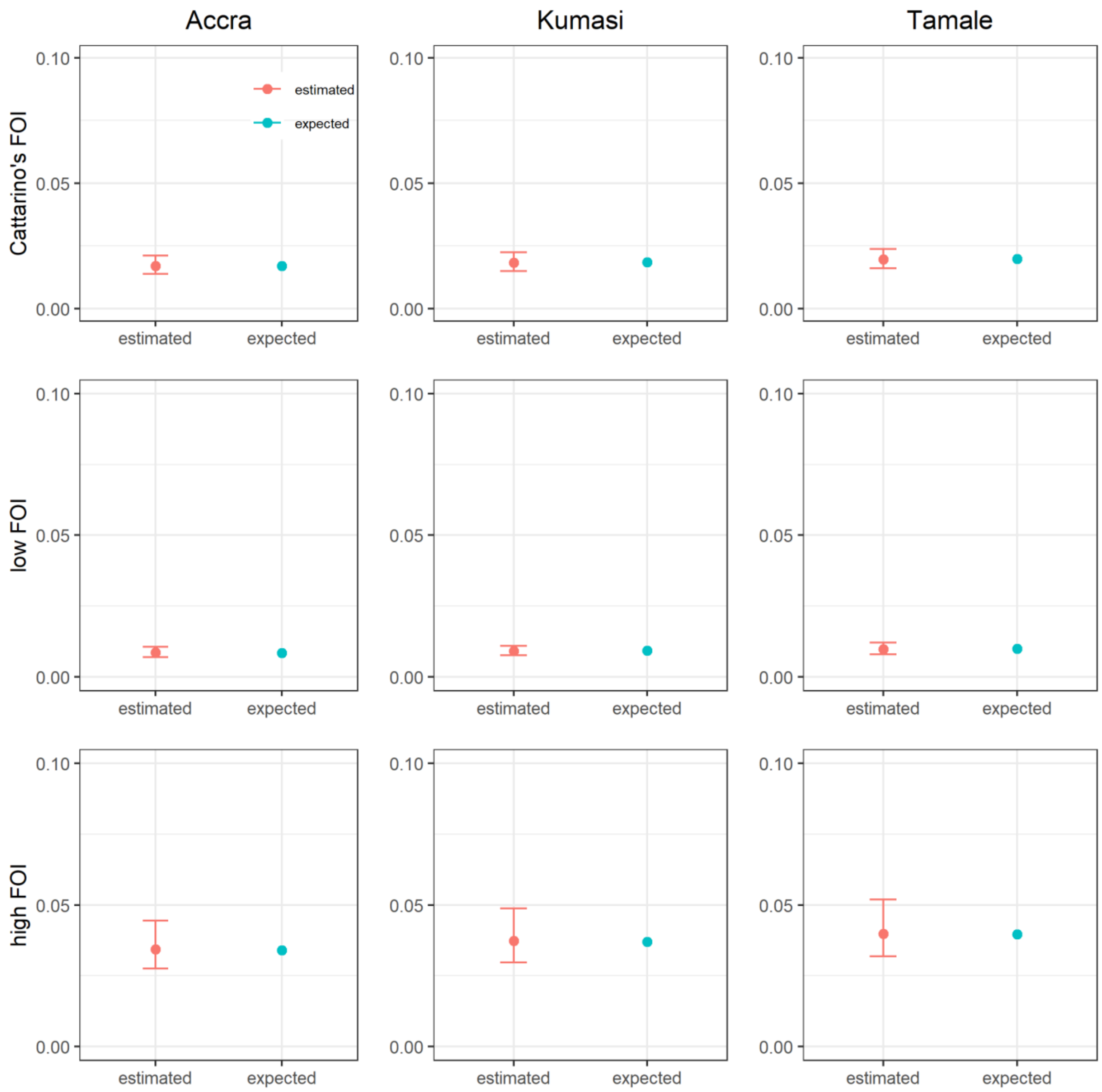

Figure C: Comparison of FOI estimates obtained with the chosen scenario vs the expected value using model 1. Each panel shows the median (point) and 95% CrI (error bar) of the FOI estimates obtained with the chosen scenario and model 1 (orange) compared to the expected FOI used to generate the simulated data (blue). The three rows represent respectively the simulated scenarios obtained with i) the FOI estimate from Cattarino et al. [3], ii) half the FOI estimate from Cattarino et al. [3] (low *FOI*) and iii) twice estimate the FOI from Cattarino et al. [3] (high *FOI*).

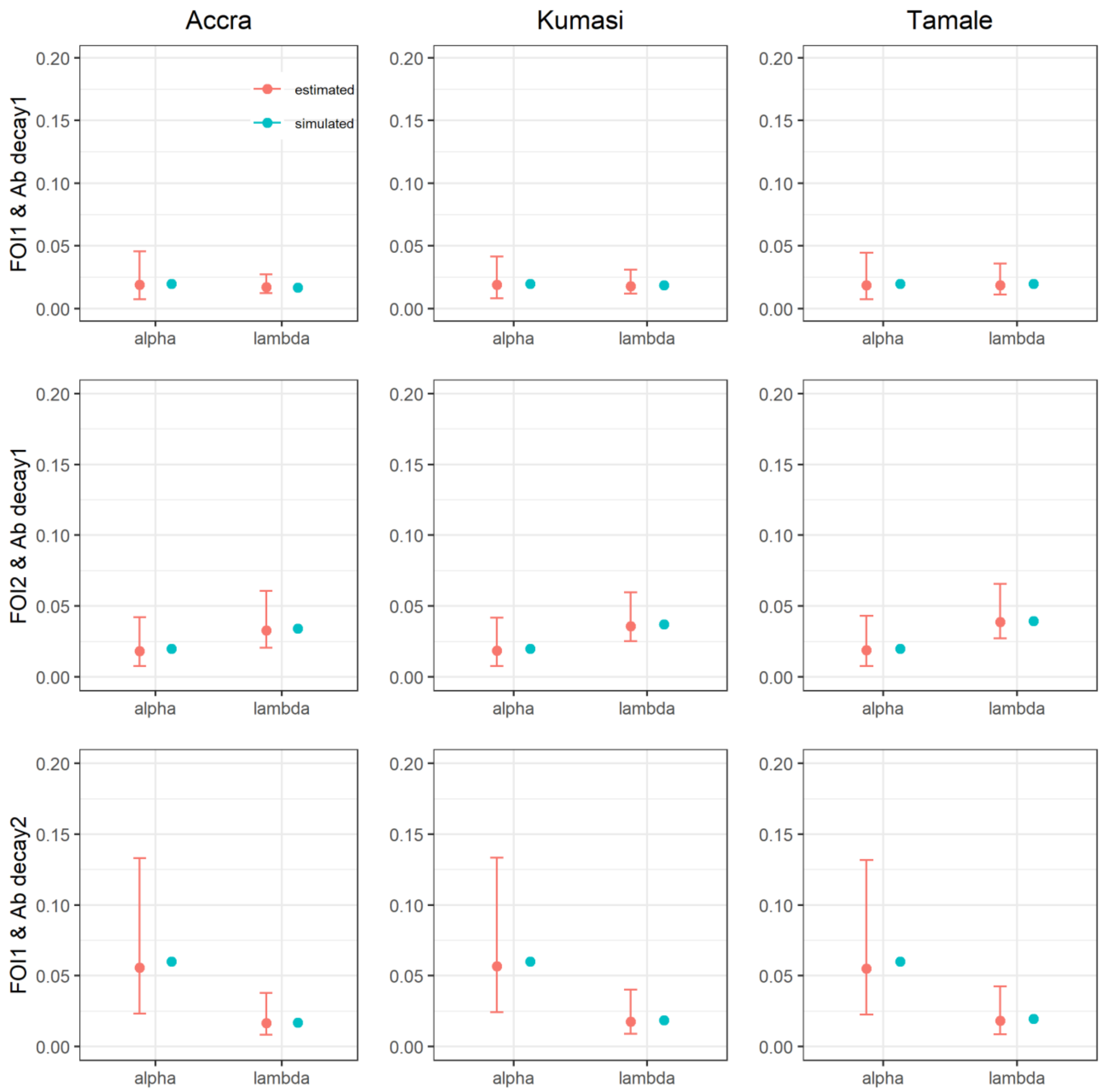

Figure D: Comparison of FOI (lambda) and decay rate (alpha) estimates obtained with the chosen scenario vs the expected value using model 2. Each panel shows the median (point) and 95% CrI (error bar) of the estimates obtained with the chosen scenario and model 2 (orange) compared to the expected estimates used to generate the simulated data (blue). The three rows represent respectively the simulated scenarios obtained with i) the FOI from Cattarino et al. [3] (*FOI1*) and a decay rate α = 0.02 (*Ab decay1*), ii) twice the FOI from Cattarino et al. [3] (*FOI2*) and α = 0.02 (*Ab decay1*), and iii) the FOI from Cattarino et al. [3] (*FOI1*) and a higher decay rate α = 0.06 (*Ab decay2*).

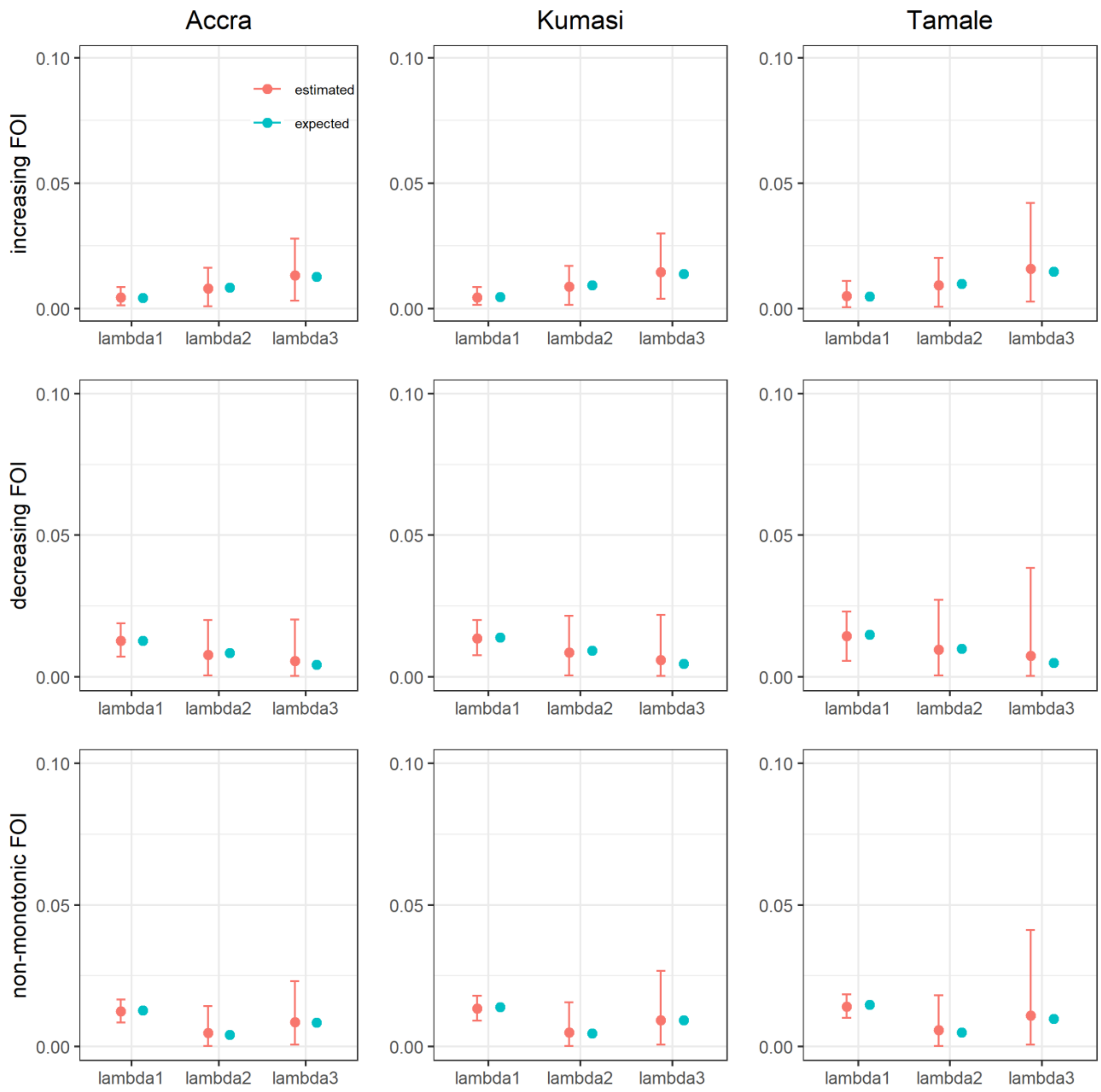

Figure E: Comparison of FOI estimates obtained with the chosen scenario vs the expected value using model 3. Each panel shows the median (point) and 95% CrI (error bar) of the FOI estimates obtained with the chosen scenario and model 3 (orange) compared to the expected FOI used to generate the simulated data (blue). The three rows represent respectively the simulated scenarios obtained with increasing, decreasing and non-monotonic FOIs.

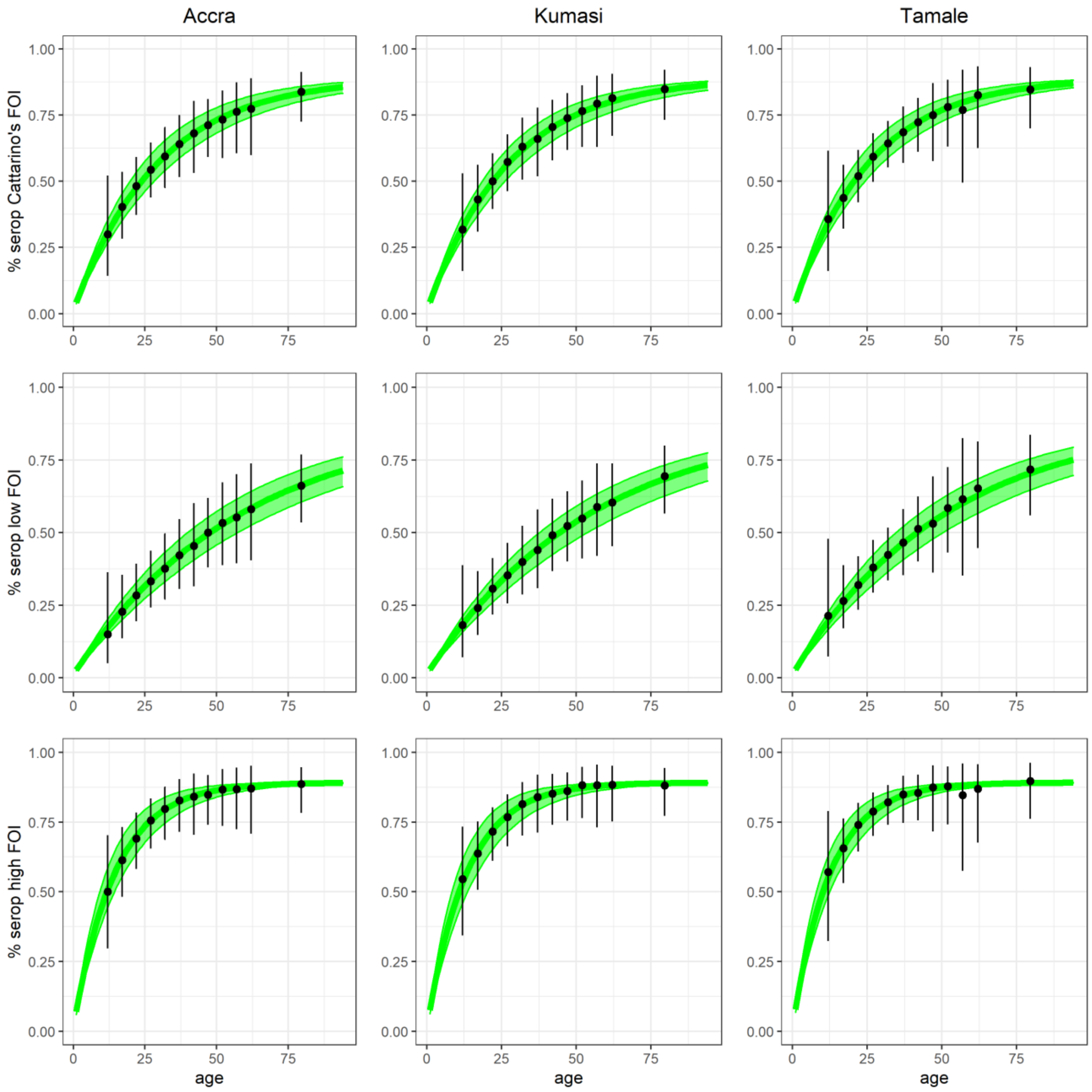

Figure F: Model fit for the three cities obtained using the chosen scenario with model 1. Each panel shows the median (line) and 95% CrI (ribbon) of the estimated seroprevalence using the chosen scenario (green). The data used for model fitting are reported in black, with the error bar representing to the exact binomial 95% CI. The three rows represent respectively the simulated data and fit obtained with i) the FOI estimate from Cattarino et al. [3], ii) half the FOI estimate from Cattarino et al. [3] (low FOI) and iii) twice the FOI estimate from Cattarino et al. [3] (high FOI).

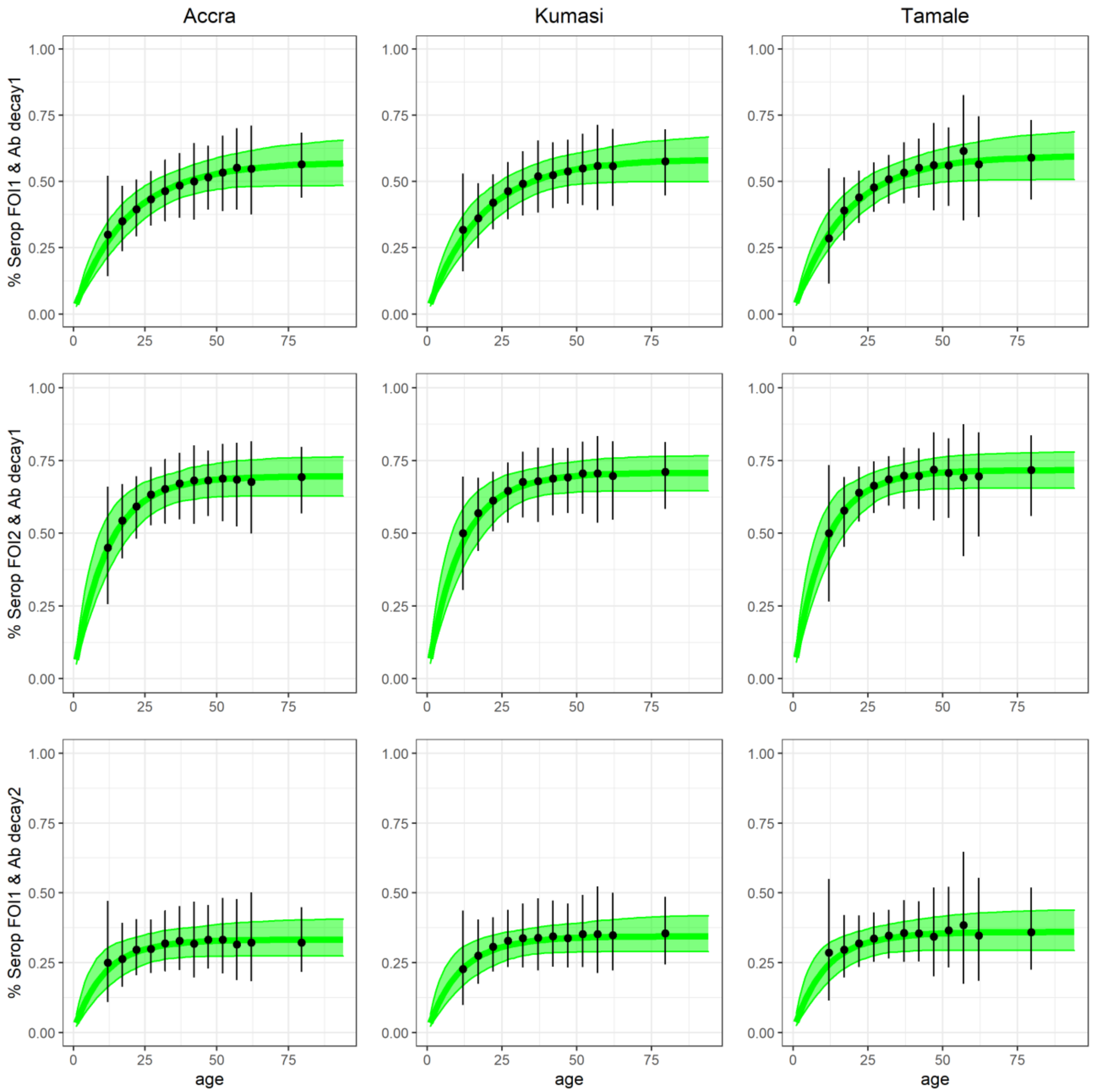

Figure G: Model fit for the three cities using the chosen scenario with model 2. Each panel shows the median (line) and 95% CrI (ribbon) of the estimated seroprevalence using the chosen scenario (green). The data used for model fitting are reported in black, with the error bar representing to the exact binomial 95% CI. The three rows represent respectively the data and fit obtained with i) the FOI from Cattarino et al. [3] (*FOI1*) and a decay rate α = 0.02 (*Ab decay1*), ii) twice the FOI from Cattarino et al. [3] (*FOI2*) and α = 0.02 (*Ab decay1*), and iii) the FOI from Cattarino et al. [3] (*FOI1*) and a higher decay rate α = 0.06 (*Ab decay2*).

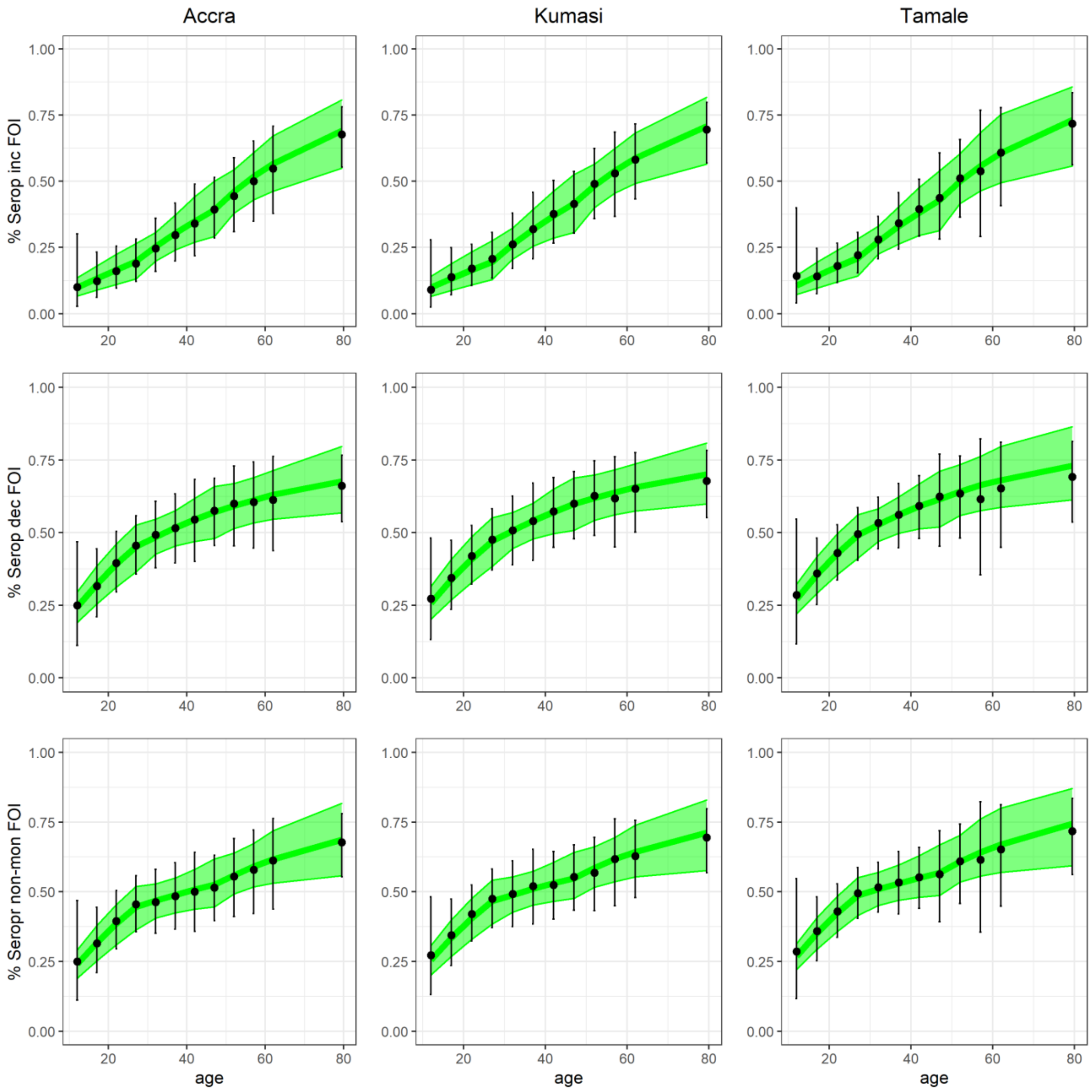

Figure H: Model fit for the three cities using the chosen scenario with model 3. Each panel shows the median (line) and 95% CrI (ribbon) of the estimated seroprevalence using the chosen scenario (green). The data used for model fitting are reported in black, with the error bar representing to the exact binomial 95% CI. The three rows represent respectively the data and fit obtained with increasing (inc), decreasing (dec) and non-monotonic (non-mon) FOIs.

Tables

Table A. Sample sizes for 5-year age categorization used in scenarios 0 to E in the three cities in Ghana.

	Accra						Kumasi						Tamale					
**Age group 5-years**	**0**	**A**	**B**	**C**	**D**	**E**	**0**	**A**	**B**	**C**	**D**	**E**	**0**	**A**	**B**	**C**	**D**	**E**
10-14	20	20	20	16	20	10	22	22	22	17	22	11	14	13	14	6	14	7
15-19	57	20	57	16	57	28	58	22	58	17	58	29	64	13	64	6	64	32
20-24	81	20	81	16	81	40	88	22	88	17	88	44	100	13	100	6	100	50
25-29	90	20	90	16	90	45	82	22	82	17	82	41	113	13	113	6	113	56
30-34	69	20	69	16	69	34	65	22	65	17	65	32	118	13	118	6	118	59
35-39	64	20	64	16	64	32	50	22	50	17	50	25	73	13	73	6	73	36
40-44	44	20	10	44	22	44	61	22	11	61	30	61	76	13	7	76	38	76
45-49	66	20	10	66	33	66	65	22	11	65	32	65	32	13	7	32	16	32
50-54	45	20	10	45	22	45	51	22	11	51	26	51	41	13	7	41	20	41
55-59	38	20	10	38	19	38	34	22	11	34	17	34	13	13	7	13	6	13
60-64	31	20	10	31	16	31	43	22	11	43	22	43	23	13	7	23	12	23
65+	62	20	10	62	31	62	59	22	11	59	30	59	39	13	7	39	20	39
**Total**	**667**	**240**	**441**	**382**	**524**	**475**	**678**	**264**	**431**	**415**	**522**	**495**	**706**	**156**	**524**	**260**	**594**	**464**

0 denotes the baseline scenario which includes all available samples; A denotes the scenario testing an equal number of samples in each age-group, using the minimum number of available samples by age-group; B denotes the scenario testing all available samples in the younger age-groups (i.e., the first half of the age-groups), and half of the minimum number of samples across the young-age groups for the older age-groups; C denotes the scenario testing all available samples for the older age-groups and half of the minimum number of samples across the old-age groups for the younger age-groups; D denotes the scenario testing all available samples for the younger age-groups and half of the available number of samples for the older age-groups and E denotes the scenario testing all available samples for the older age-groups and half of the available number of samples for the younger age-groups

Table B. Sample sizes for 5-year age categorisation used in scenarios 0-E for Accra.

Age group 5 years	Scenario 0	Scenario A	Scenario B	Scenario C	Scenario D	Scenario E
**10-14**	20	20	20	16	20	10
**15-19**	57	20	57	16	57	28
**20-24**	81	20	81	16	81	40
**25-29**	90	20	90	16	90	45
**30-34**	69	20	69	16	69	34
**35-39**	64	20	64	16	64	32
**40-44**	44	20	10	44	22	44
**45-49**	66	20	10	66	33	66
**50-54**	45	20	10	45	22	45
**55-59**	38	20	10	38	19	38
**60-64**	31	20	10	31	16	31
**65+**	62	20	10	62	31	62
**Total**	667	240	441	382	524	475

Table C. Sample sizes for 5-year age categorisation used in scenarios 0-E for Kumasi.

Age group 5 years	Scenario 0	Scenario A	Scenario B	Scenario C	Scenario D	Scenario E
**10-14**	22	22	22	17	22	11
**15-19**	58	22	58	17	58	29
**20-24**	88	22	88	17	88	44
**25-29**	82	22	82	17	82	41
**30-34**	65	22	65	17	65	32
**35-39**	50	22	50	17	50	25
**40-44**	61	22	11	61	30	61
**45-49**	65	22	11	65	32	65
**50-54**	51	22	11	51	26	51
**55-59**	34	22	11	34	17	34
**60-64**	43	22	11	43	22	43
**65+**	59	22	11	59	30	59
**Total**	678	264	431	415	522	495

Table D. Sample sizes for 5-year age categorisation used in scenarios 0-E for Tamale.

Age group 5 years	Scenario 0	Scenario A	Scenario B	Scenario C	Scenario D	Scenario E
**10-14**	14	13	14	6	14	7
**15-19**	64	13	64	6	64	32
**20-24**	100	13	100	6	100	50
**25-29**	113	13	113	6	113	56
**30-34**	118	13	118	6	118	59
**35-39**	73	13	73	6	73	36
**40-44**	76	13	7	76	38	76
**45-49**	32	13	7	32	16	32
**50-54**	41	13	7	41	20	41
**55-59**	13	13	7	13	6	13
**60-64**	23	13	7	23	12	23
**65+**	39	13	7	39	20	39
**Total**	706	156	524	260	594	464

Table E. Sample sizes for 10-year age categorisation used in scenarios 0-E for Accra.

Age group 10 years	Scenario 0	Scenario A	Scenario B	Scenario C	Scenario D	Scenario F
**10-19**	77	20	77	42	77	38
**20-29**	171	20	171	42	171	86
**30-39**	133	20	133	42	133	66
**40-49**	110	20	38	110	55	110
**50-59**	83	20	38	83	42	83
**≥60**	93	20	38	93	46	93
**Total**	667	120	495	412	524	476

Table F. Sample sizes for 10-year age categorisation used in scenarios 0-E for Kumasi.

Age group 10 years	Scenario 0	Scenario A	Scenario B	Scenario C	Scenario D	Scenario E
**10-19**	80	22	80	42	80	40
**20-29**	170	22	170	42	170	85
**30-39**	115	22	115	42	115	58
**40-49**	126	22	40	126	63	126
**50-59**	85	22	40	85	42	85
**≥60**	102	22	40	102	51	102
**Total**	678	132	485	439	521	496

Table G. Sample sizes for 10-year age categorisation used in scenarios 0-E for Tamale.

Age group 10 years	Scenario 0	Scenario A	Scenario B	Scenario C	Scenario D	Scenario E
**10-19**	78	13	78	27	78	39
**20-29**	213	13	213	27	213	106
**30-39**	191	13	191	27	191	96
**40-49**	108	13	39	108	54	108
**50-59**	54	13	39	54	27	54
**≥60**	62	13	39	62	31	62
**Total**	706	78	599	305	594	465

Table H. Age-specific samples sizes for the 5-years age categorisation with the selected scenario.

Age group 5-years	Accra	Kumasi	Tamale
**Scenario**	**B**	**D**	**B**
**10-14**	20	22	14
**15-19**	57	58	64
**20-24**	81	88	100
**25-29**	90	82	113
**30-34**	69	65	118
**35-39**	64	50	73
**40-44**	10	30	7
**45-49**	10	32	7
**50-54**	10	26	7
**55-59**	10	17	7
**60-64**	10	22	7
**65+**	10	30	7
**Total**	441	522	524

Table K. Age-specific samples sizes for the 10-years age categorisation with the selected scenario.

Age group 10-years	Accra	Kumasi	Tamale
**Scenario**	**B**	**D**	**D**
**10-19**	77	80	78
**20-29**	171	170	213
**30-39**	133	115	191
**40-49**	38	63	54
**50-59**	38	42	27
**≥60**	38	51	31
**Total**	495	521	594

Table J. City-specific lambda FOI estimates under model 3.

Time-varying FOI	Accra	Kumasi	Tamale	Description
**Increasing FOI**	$\lambda_1 = 0.0042$	$\lambda_1 = 0.0046$	$\lambda_1 = 0.0049$	Acts on all age-groups
	$\lambda_2 = 0.0085$	$\lambda_2 = 0.0092$	$\lambda_2 = 0.0099$	Acts on age-groups > 30 years
	$\lambda_3 = 0.0127$	$\lambda_3 = 0.0139$	$\lambda_3 = 0.0148$	Acts on age-groups > 50 years
**Decreasing FOI**	$\lambda_1 = 0.0127$	$\lambda_1 = 0.0139$	$\lambda_1 = 0.0148$	Acts on all age-groups
	$\lambda_2 = 0.0085$	$\lambda_2 = 0.0092$	$\lambda_2 = 0.0099$	Acts on age-groups > 30 years
	$\lambda_3 = 0.0042$	$\lambda_3 = 0.0046$	$\lambda_3 = 0.0049$	Acts on age-groups > 50 years
**Non-monotonic FOI**	$\lambda_1 = 0.0127$	$\lambda_1 = 0.0139$	$\lambda_1 = 0.0148$	Acts on all age-groups
	$\lambda_2 = 0.0042$	$\lambda_2 = 0.0046$	$\lambda_2 = 0.0049$	Acts on age-groups > 30 years
	$\lambda_3 = 0.0085$	$\lambda_3 = 0.0092$	$\lambda_3 = 0.0099$	Acts on age-groups > 50 years

Table I. Total number of samples in Ghana according to the selected scenario compared to all available samples.

	Accra	Kumasi	Tamale
**Scenario**	B	D	B
**Total available**	667	678	706
**Total selected**	441	522	524

Two-step testing approach

Example distribution of the second half of the samples in the two-step testing process
In this section we show how it is possible to adjust the distribution of the samples if a too high or too low FOI was assumed in the initial phase of the study. Specifically, we show how to adjust the sample distribution in the hypothetical scenario where (i) the true FOI estimate was half of the initially assumed FOI value (this scenario was applied to the sample sizes collected in Accra) and (ii) the true FOI estimate was twice the initially assumed FOI value (this scenario was applied to the sample sizes collected in Kumasi).
Using the FOI estimate obtained from the interim analysis of half of the samples, we repeated the simulation study performed initially. In Fig K, we compared the chosen scenarios selected based on the interim analysis (orange) and the initial study (green) for the two locations.

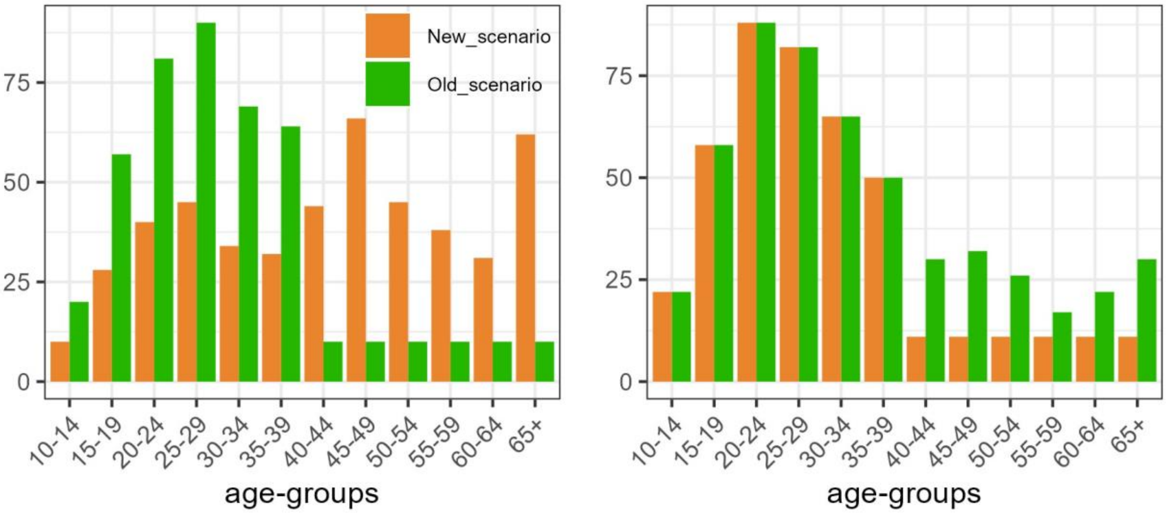

Figure K: Comparison of sample distributions obtained using the Cattarino et al. FOI estimates (“Old_scenario”, green) vs the sample distribution obtained if assuming half of the FOI value (left) and twice the FOI value (right) (“New_scenario”, orange).

It is hence possible to distribute the remaining samples available to match the newly chosen distribution (in orange in Fig K) by (1) computing the total number of samples per age-group using the overall sample sizes available under the new optimal scenario (2) calculating the difference between the age-distribution in step (1) and the age-distribution of the samples already tested for the interim analysis (Fig J).

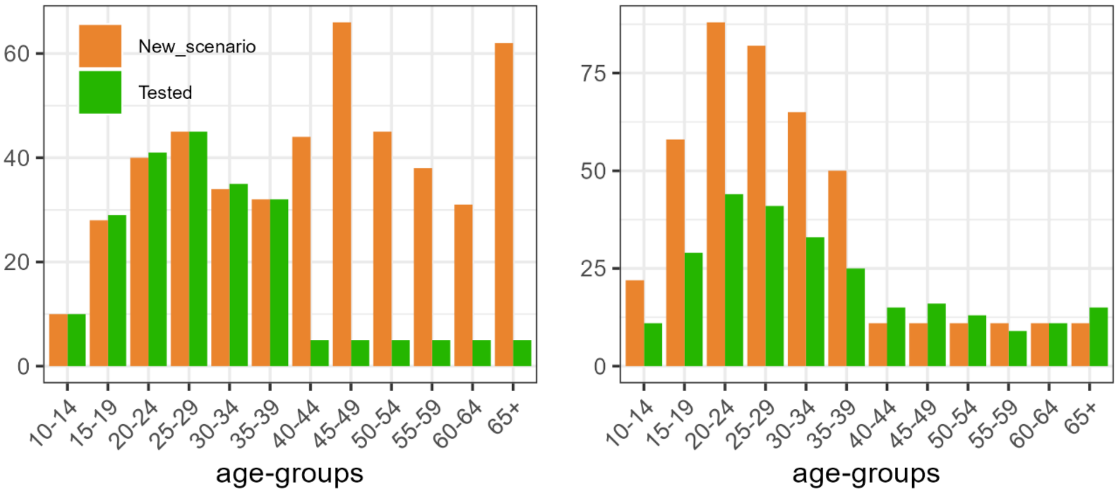

Figure J: Comparison of sample distributions obtained using new FOI estimates (“New_scenario”, orange) vs the sample distribution from phase I under the old optimal scenario (“Tested”, green).

This difference represents the age-distribution needed for the second and final phase of the testing. If in an age-group more samples than those identified in the newly chosen scenario had already been tested in phase 1, we set the difference equal to zero (i.e., we do not add any more tests in that age-group). In the event that the samples sizes of the newly chosen scenario exceed the sample sizes of the old scenario, it is possible to rescale the new age-distribution by the number of samples available if the purchase of new tests is not possible. Fig I shows the final distribution achieved at the end of the study with the re-distribution method described in this section (*Final_test*) with the newly chosen scenario identified in the interim analysis (*New_scenario*).

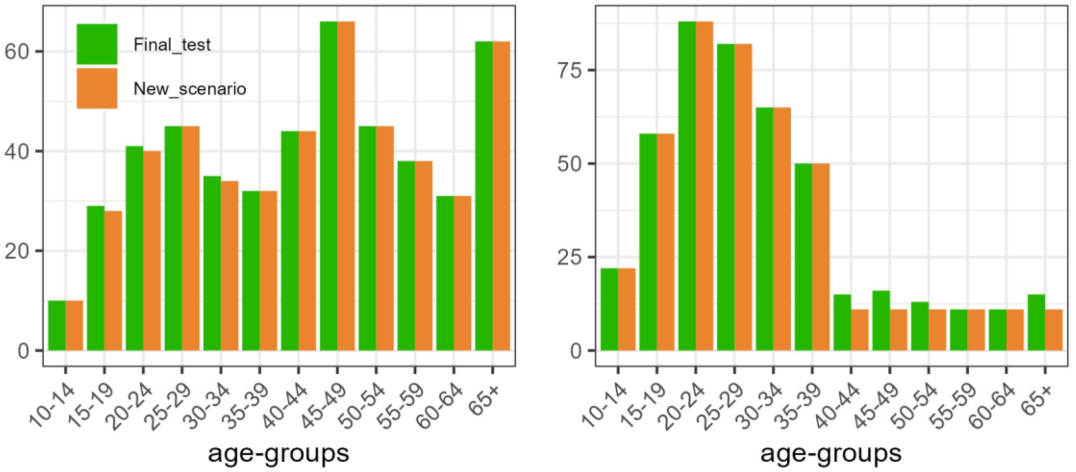

Figure I: Comparison between actual samples distribution achieved ("Final_test") **vs the optimal distribution** identified in the interim analysis ("New_scenario").

Fig L compares the FOI estimates obtained with the old scenario (selected from step I of the analysis) and the new scenario (selected from step II of the analysis) with the assumed FOI and true FOI values (respectively, half of the assumed FOI in Accra and twice of the assumed FOI in Kumasi). Fig. L shows that by changing the sample size distributions during phase II, it is possible to obtain unbiased FOI estimates that closely capture the true FOI estimate.

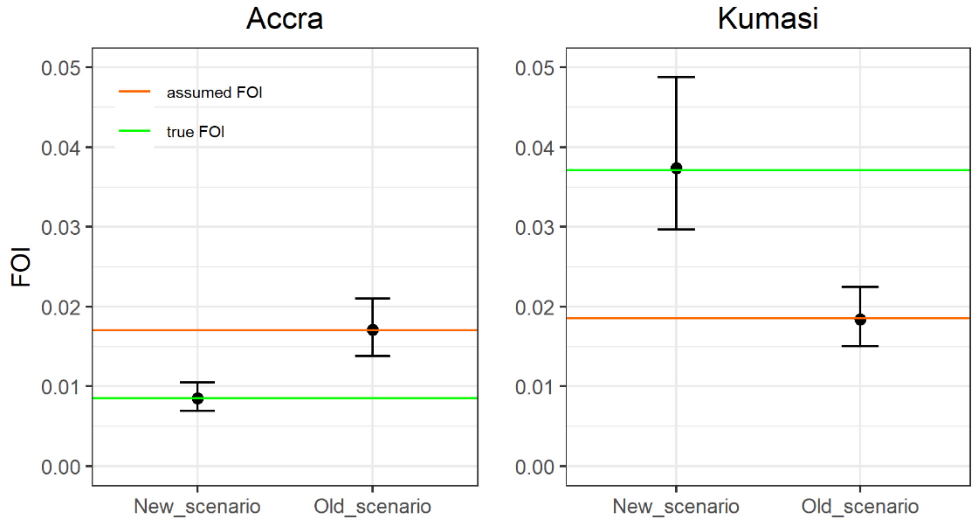

Figure L: Comparison of FOI estimates obtained after phase I ("Old_scenario") and after phase II ("New_scenario") vs the assumed and true FOI values. Median (point) and 95% CrI (error bar) of the FOI estimates obtained after phase I ("Old_scenario") and phase II ("New_scenario") as compared to the assumed FOI (orange line) and true FOI (green line) in the hypothetical scenarios explored.
