# Supplementary material for: A simulation-based method to inform serosurvey design for estimating the force of infection using existing blood samples
Source: PLoS Comput Biol. 2023 Nov 27;19(11):e1011666. doi: 10.1371/journal.pcbi.1011666 (PMC10727435; doi:10.1371/journal.pcbi.1011666)
Supplement: Attachment — Submitted filename: Point_by_point_response_to_reviewers_7Aug_final.docx [file pcbi.1011666.s003.docx]

**Point-by-point response to reviewers**

**A simulation-based method to inform serosurvey designs for estimating the force of infection using existing blood samples**

Anna Vicco, Clare McCormack, Belen Pedrique, John H. Amuasi, Anthony Afum-Adjei Awuah, Christian Obirikorang, Nicole S. Struck, Eva Lorenz, Jürgen May, Isabela Ribeiro, Gathsaurie Neelika Malavige, Christl A. Donnelly, Ilaria Dorigatti

**Reviewer #1: Vicco et al. describe a simulation approach for determining sample sizes and strategies when estimating the force of infection using serocatalytic models. The study focuses on estimating dengue FOI from age-stratified blood samples collected during the SARS-CoV-2 pandemic from 3 cities in Ghana; the crux of the problem being that the age-distribution of the samples was not optimised for dengue surveillance, and not all samples could be retested. The authors used a simulation-recovery approach to compare the bias, coverage and uncertainty of FOI estimates when using different subsets of the full sample set. The authors suggest that this approach could be used to optimize serosurvey design for other pathogen systems and propose a conceptual workflow for testing serum samples in two stages for more efficient test allocation.

The authors did a good job of explaining their sample selection method which will help researchers inform their serosurveys. The code is also available and very well documented, which is great. However, while technically sound, we feel that the study is very specific to their sample set and makes many simplifying assumptions. It might be worthwhile exploring how the optimal sampling design varies under different assumptions (waning immunity, varying force of infection, different mean age of infection, etc.) to demonstrate how this framework would work for different sample availabilities, epidemiology, FOIs etc. This would strengthen the author’s claim that this can be adapted to different pathogens and demonstrate how these findings are dengue-specific.**

Thank you for the positive feedback and constructive suggestions. Our study was indeed very specific to the locations and virus that motivated the study, but we appreciate the need to further explore the robustness of the methods under different assumptions and we have extended the scenarios and the simulation framework to strengthen our conclusions, as shown in detail below.

**Major comments
1. Lack of literature context
The introduction is a great overview of dengue epidemiology and vaccine development. However, given that the paper is about simulation-guided serosurveys, there is a lack of literature review on studies that already exist in this space, and most of the information on dengue is not relevant. We are also sceptical of the claims of novelty here, as simulation-recovery is a standard tool for power calculations in seroepidemiology. The following studies address the task of optimizing serosurvey design for estimating epidemic dynamics (including simulations), arguably more thoroughly than the present study:

1. Vinh & Boni. Epidemics 2015 doi: 10.1016/j.epidem.2015.02.005
2. Sepúlveda et al. Malar J 2015 DOI: 10.1186/s12936-015-1050-3
3. Blaizot et al. BMC Medical Research Methodology 2019 https://doi.org/10.1186/s12874-019-0692-1
4. Larremore et al. eLife 2021 https://doi.org/10.7554/eLife.64206
5. The review article by Cutts & Handson https://doi.org/10.1111/tmi.12737 also has some good references.

Granted, Vicco et al. are investigating serocatalytic models, whereas most of these studies are focused on fitting compartmental models. I would suggest substantially reframing the introduction and discussion to place the study in its correct context, rather than focusing on dengue epidemiology which is arguably only incidental to this study.**

We agree that the original introduction could have been better placed in the context of simulation studies and we thank the reviewer for the suggested references. We have re-written the introduction to better contextualise our study in the light of the existing literature.

**Summary of changes:**

Line 48-89: we have rewritten the introduction as suggested, which now reads as follows:

*“Dengue virus (DENV) is one of the most rapidly spreading vector-borne diseases worldwide and places a large burden on public health [1]. In the last 50 years, dengue incidence has increased more than 30-fold [2], with recent estimates suggesting that up to 105 million people are infected each year [3]. Due to ongoing changes in climate, demography, socioeconomic structure and growing urbanization [4], there are concerns that the geographical range of dengue will expand in the coming decades.*

*Estimates suggest this potential geographic expansion may affect Sub-Saharan Africa in particular [5-7], a region where the burden of dengue remains poorly understood and is highly uncertain [8, 9]. There are also key knowledge gaps on the rates of asymptomatic infection [4, 10] and misclassification [2]. In addition, the lack of a strong surveillance system and insufficient infrastructure, have been identified as key challenges for arbovirus surveillance and disease control across the African region [11].*

*Cross-sectional serological surveys, which involve the collection of sera samples and the subsequent analysis of antibody level in the sera [12], are the gold standard for assessing individual-level exposure and for understanding the historical circulation of dengue at the population-level [13]. Age-stratified serological surveys provide the necessary data to reconstruct the immunity profile of the population, i.e., the extent of dengue exposure as a function of age, which in turn can be used to estimate the force of infection (FOI), defined as the per capita rate at which susceptible individuals become infected [10, 14], the burden of infection transmission intensity and the impact of interventions [3].*

*However, several challenges, including the need of substantial resources and infrastructure, have posed limitations on the number of serosurveys conducted in low-income countries [12]. In Africa for example, only 17 age-stratified dengue seroprevalence surveys have been conducted to date [3, 15]. Optimizing the serosurvey design, particularly the number of samples to be tested, is critical to promoting the use of these surveys, especially when resources are limited. While conducting new seroprevalence surveys provides the opportunity to target optimal age-groups for estimating epidemiological parameters, it also requires extensive resources, including time and operational capacity. In some cases these requirements can be mitigated by leveraging existing blood samples.*

*Simulation-based analyses are a powerful tool for informing serosurvey design as they enable the testing of different sampling scenarios, which in turn can help determine sample sizes targeted to the outcomes of interest [16-18]. For example, reversible catalytic model have been used to optimize the sample sizes required to accurately estimate the seroconversion rate to malaria [16], compartmental dynamic transmission models have been developed to identify the optimal age distribution of blood samples for different pathogens and outcomes of interest [17] and during the COVID-19 pandemic, Bayesian methods were developed to propagate the uncertainty of imperfect testing and determine optimal sample size to minimize the uncertainty in the seroprevalence estimates [18].*

*In this study, we developed methods to inform serosurvey design when leveraging existing blood samples. We use simple catalytic models to develop a new simulation-based framework for identifying the optimal number and age distribution of available blood samples to obtain accurate setting-specific dengue FOI estimates whist optimising resources and at the same time accounting for imperfect test sensitivity and specificity. The methods described in this study have been used to inform the design of serosurveys aimed to reconstruct dengue FOI estimates in three locations in Ghana [19], utilising blood samples previously collected for SARS-CoV-2 surveillance. Below we provide evidence of how the simulation framework developed in this study can be readily adopted for estimating the FOI of other infectious pathogens across different transmission settings.”*

**2. Overly simple serocatalytic model
The serocatalytic model seems more suitable for a simple, single-variant pathogen rather than one as complex as dengue, given the epidemiology described in the introduction. For example, the authors refer to the literature on dengue serotype interactions (cross-reactivity in measured antibodies and temporary cross-protection between serotypes), but the model in equation 1 ignores these interactions and assumes that the force of infection is simply additive across serotypes (nλ). With this formulation, the n is irrelevant as it is just a fixed constant – we cannot disentangle the relative contribution of each serotype nor their cross-reactivity, and thus the estimated FOI is just an aggregate of all dengue activity (and there is complete cross-protection between serotypes). Other standard additions to the serocatalytic model are seroreversion and time-varying FOI, which could be explored in the simulation framework – it would be interesting to understand how the optimal sample set differs depending on the complexity of the fitted model. I did appreciate the inclusion of test sensitivity and specificity, though the robustness to different test accuracies could also be demonstrated. I would encourage the authors to explore these more complex models, both to demonstrate the application of this framework to different pathogens and to better capture the epidemiology of dengue.**

We thank the reviewer for the suggestion. The estimated FOI is an aggregated measure of dengue activity because this is what is measured with the IgG ELISA assay. We multiplied the estimates provided n Cattarino et al., which represent per-serotype FOI estimates, by the number of serotypes in circulation in Ghana according to the literature. In the revised version of the manuscript, we have extended our analysis to include: (i) higher and lower FOI values in the time constant catalytic model (model 1), (ii) waning of immunity (model 2), and (iii) time-varying FOIs (model 3). Also for models 2 and 3, we tested the sensitivity of our results on the assumed FOI values, alternative rates of antibody decay and different patterns of transmission in model 3, including increasing, decreasing and non-monotonic FOIs. All additions have been included in the shared R code too.

**Summary of changes:**

- Line 167-20: addition of the sensitivity analysis description. In more details, we added:

*“Alternative categorisation used to report the age-specific seroprevalence*

*In a sensitivity analysis, we ran the simulation study using 10-year age categories (Fig S2) (instead of 5-year age categories as done in the main analysis) to explore the effect that reporting the age-specific seroprevalence over wider age-groups has on the precision of the FOI estimates.*

*Alternative serocatalytic model formulations*

*To adapt our framework to different pathogens and settings, we implemented the simulation framework for the case where antibody protection decays over time at a yearly rate α (model 2) [10]****.*** *In this scenario, the seroprevalence can be modelled as shown in Eq. 8.*

$z\left( a_{i} \right)=\frac{\lambda}{\lambda+ \alpha}\left[ 1- e^{{- n a}_{i}(\lambda+ \alpha)} \right]$	*(8)*

*We also explored scenarios with time-varying FOIs (model 3) [25]. In our simulation study we assumed that the FOI changed in a stepwise fashion over time (Table S10), with individuals 0-29 years old (age-groups i < 5) exposed solely to* $\lambda_{1},$*individuals 30-49 years old (age-groups* $5\leq i\leq8$*) exposed to* $\lambda_{1}$ *during the last 30 years of life and to* $\lambda_{2}$ *during the first 20 years of life and individuals 50+ years of age (age-groups i > 8) exposed to* $\lambda_{1}$ *during the last 30 years of life,* $\lambda_{2}$ *during the middle 20 years of life and* $\lambda_{3}$ *for the earlier years of their life.* *In this case the age-stratified seroprevalence can be described as shown in Eq. 9:*

$z{(a}_{i})=\left\{ \begin{matrix} 1-e^{- n\lambda_{1}a_{i}}, & i <5 \\ 1-e^{- n\left[ \lambda_{1}a_{4} + {\lambda_{2}(a}_{i}-a_{4}) \right]}, & 5\leq i\leq8 \\ 1-e^{- n\left[ \lambda_{1}a_{4} + {\lambda_{2}(a}_{8}-a_{4})+{\lambda_{3}(a}_{i}-a_{8}) \right]}, & i>8 \end{matrix} \right.$	*(9)*

*where a_i_ is the midpoint of age-group i,* $a_{4}$ *is the mid- point of age-group i = 4 (25-29 years old), and* $a_{8}$ *is the mid-point of age-group i = 8 (45-49 years old).*

*We used models 2 and 3 to generate simulated datasets, and then used the corresponding catalytic model (respectively model 2 and 3) and the likelihood defined in Eq. 7 to reconstruct the posterior distribution of the FOI λ and decay rate* $\alpha$ *in model 2 and of the three FOIs* $\lambda_{1}$*,* $\lambda_{2}$ *and* $\lambda_{3}$ *in model 3. We assumed a normal distribution with mean 0 and standard deviation of 1 for the lambda parameters in both model 2 and 3, and a normal distribution with mean 0.02 (as suggested in the literature [10]) and standard deviation of 0.5 for the decay rate in model 2. We sampled the parameters on the log scale to improve the exploration of the parameter space.*

*Alternative assumptions on the magnitude of the FOI and decay rate*

*In model 1, we tested three different FOI values: 1)* $\lambda$ *taken from Cattarino et al. [3]; 2) twice the value taken from Cattarino et al. [3]; and 3) half the value taken from Cattarino et al. [3], as shown in Fig S3.*

*In model 2, we applied our framework to three alternative scenarios exploring different FOI and decay rate values: scenario 1)* $\lambda$ *taken from Cattarino et al. [3] and a decay rate α = 0.02 from Imai et al. [10]; scenario 2)* $\lambda$ *equal to twice the estimate in Cattarino et al. [3] and α = 0.02; and scenario 3)* $\lambda$ *taken from Cattarino et al. [3] and a higher decay rate α = 0.06. Table 1 and Fig S4 show the values of* $\lambda$ *and α used in the three scenarios.*

*In model 3 we tested three different patterns of transmission, with increasing, decreasing, and non-monotonic FOIs as shown in Fig S5. In the selection of the optimal sampling scenario, we calculated the average bias, coverage and uncertainty over the three FOIs.”*

- We moved the previous Table 1 to the SI and included a new Table 1, summarising the results obtained with model 1, 2 and 3 as shown below:

***Table 1. Summary of the accuracy metrics obtained for Ghana with 5-year age category under the optimal scenarios across models and assumptions.***

| *Model* | *Type* | *Parameter values* | *Median age of first infection* | *City* | *Bias (95% CrI)* | *Coverage (95% CI)* | *Uncertainty (95% CrI)* | *Scenario* | *N samples* |
| --- | --- | --- | --- | --- | --- | --- | --- | --- | --- |
| *1* | *Cattarino’s FOI* | $\lambda=0.017$ | *29* | *Accra* | *5.2 (0.3, 17.2)* | *95.2 (93.7, 96.3)* | *0.005 (0.004, 0.006)* | ***B*** | *441* |
| *1* | *Cattarino’s FOI* | $\lambda= 0.020$ | *25* | *Tamale* | *4.3 (0.2, 14.8)* | *95.8 (94.4, 96.9)* | *0.005 (0.005, 0.006)* | ***B*** | *524* |
| *1* | *Cattarino’s FOI* | $\lambda= 0.019$ | *26* | *Kumasi* | *4.7 (0.2, 16.4)* | *94.4 (92.8, 95.7)* | *0.005 (0.004, 0.006)* | ***D*** | *522* |
| *1* | *High FOI* | $\lambda=0.034$ | *15* | *Accra* | *5.3 (0.2, 21.2)* | *95 (93.5, 96.2)* | *0.012 (0.009, 0.016)* | ***B*** | *441* |
| *1* | *High FOI* | $\lambda=0.040$ | *13* | *Tamale* | *5.7 (0.3, 21.9)* | *94.3 (92.7, 95.6)* | *0.014 (0.01, 0.02)* | ***B*** | *524* |
| *1* | *High FOI* | $\lambda=0.038$ | *13* | *Kumasi* | *5.8 (0.4, 20.3)* | *96.3 (94.9, 97.3)* | *0.014 (0.011, 0.019)* | ***B*** | *431* |
| *1* | *Low FOI* | $\lambda=0.009$ | *59* | *Accra* | *5.1 (0.2, 17)* | *94.6 (93, 95.8)* | *0.003 (0.002, 0.003)* | ***E*** | *475* |
| *1* | *Low FOI* | $\lambda= 0.010$ | *50* | *Tamale* | *5.4 (0.3, 16.7)* | *94.7 (93.1, 95.9)* | *0.003 (0.003, 0.003)* | ***E*** | *464* |
| *1* | *Low FOI* | $\lambda= 0.010$ | *53* | *Kumasi* | *4.4 (0.3, 14.4)* | *93.9 (92.2, 95.2)* | *0.002 (0.002, 0.003)* | ***0*** | *678* |
| *2* | *Cattarino’s FOI, baseline decay* | $\lambda=0.017$  $\alpha=0.02$ | *29* | *Accra* | *7.8 (0.3, 28.3)* | *99.5 (98.8, 99.8)* | *0.012 (0.008, 0.02)* | ***B*** | *441* |
| *2* | *Cattarino’s FOI, baseline decay* | $\lambda=0.020$  $\alpha=0.02$ | *25* | *Tamale* | *9.8 (0.6, 30)* | *98.7 (97.8, 99.2)* | *0.015 (0.009, 0.025)* | ***E*** | *464* |
| *2* | *Cattarino’s FOI, baseline decay* | $\lambda=0.019$  $\alpha=0.02$ | *26* | *Kumasi* | *10.5 (0.4, 32.5)* | *99.5 (98.8, 99.8)* | *0.016 (0.01, 0.026)* | ***A*** | *264* |
| *2* | *High FOI, baseline decay* | $\lambda=0.034$  $\alpha=0.02$ | *15* | *Accra* | *11.1 (0.5, 36)* | *98.7 (97.8, 99.2)* | *0.031 (0.019, 0.052)* | ***A*** | *240* |
| *2* | *High FOI, baseline decay* | $\lambda=0.040$  $\alpha= 0.02$ | *13* | *Tamale* | *9.2 (0.4, 27.3)* | *99.4 (98.7, 99.7)* | *0.03 (0.018, 0.051)* | ***B*** | *524* |
| *2* | *High FOI, baseline decay* | $\lambda=0.038$  $\alpha=0.02$ | *13* | *Kumasi* | *9.2 (0.4, 27.8)* | *98.8 (97.9, 99.3)* | *0.026 (0.016, 0.045)* | ***B*** | *431* |
| *2* | *Cattarino’s FOI, high decay* | $\lambda=0.017$  $\alpha=0.06$ | *29* | *Accra* | *13.1 (0.9, 46.8)* | *99.9 (99.4, 100)* | *0.025 (0.015, 0.037)* | ***A*** | *240* |
| *2* | *Cattarino’s FOI, high decay* | $\lambda=0.020$  $\alpha=0.06$ | *25* | *Tamale* | *14.4 (0.5, 40.8)* | *100 (99.6, 100)* | *0.031 (0.021, 0.043)* | ***C*** | *260* |
| *2* | *Cattarino’s FOI, high decay* | $\lambda=0.019$  $\alpha=0.06$ | *26* | *Kumasi* | *13.6 (0.8, 42.9)* | *99.5 (98.8, 99.8)* | *0.025 (0.015, 0.038)* | ***A*** | *264* |
| *3* | *Decreasing FOI* | $\lambda_{1}=0.013$  $\lambda_{2}=0.009$  $\lambda_{3}=0.004$ | *24* | *Accra* | *23 (1.3, 171.4)* | *97.6 (96.5, 98.4)* | *0.013 (0.008, 0.023)* | ***C*** | *382* |
| *3* | *Decreasing FOI* | $\lambda_{1}=0.015$  $\lambda_{2}=0.010$  $\lambda_{3}=0.005$ | *21* | *Tamale* | *27.7 (0.9, 253)* | *97.8 (96.7, 98.5)* | *0.018 (0.011, 0.046)* | ***C*** | *260* |
| *3* | *Decreasing FOI* | $\lambda_{1}=0.014$  $\lambda_{2}=0.009$  $\lambda_{3}=0.005$ | *22* | *Kumasi* | *23.8 (1, 172)* | *97.7 (96.6, 98.5)* | *0.014 (0.008, 0.024)* | ***C*** | *415* |
| *3* | *Increasing FOI* | $\lambda_{1}=0.004$  $\lambda_{2}=0.009$  $\lambda_{3}=0.013$ | *38* | *Accra* | *22.3 (1, 72.6)* | *95.4 (93.9, 96.5)* | *0.011 (0.004, 0.022)* | ***C*** | *382* |
| *3* | *Increasing FOI* | $\lambda_{1}=0.005$  $\lambda_{2}=0.010$  $\lambda_{3}=0.015$ | *32* | *Tamale* | *27.5 (1.7, 89.4)* | *96.6 (95.3, 97.6)* | *0.015 (0.006, 0.039)* | ***C*** | *260* |
| *3* | *Increasing FOI* | $\lambda_{1}=0.005$  $\lambda_{2}=0.009$  $\lambda_{3}=0.014$ | *35* | *Kumasi* | *19.2 (0.9, 68.2)* | *95.5 (94, 96.6)* | *0.012 (0.004, 0.023)* | ***C*** | *415* |
| *3* | *Non-monotonic FOI* | $\lambda_{1}=0.013$  $\lambda_{2}=0.004$  $\lambda_{3}=0.009$ | *29* | *Accra* | *18.6 (0.7, 119.4)* | *98 (96.9, 98.7)* | *0.011 (0.005, 0.023)* | ***E*** | *475* |
| *3* | *Non-monotonic FOI* | $\lambda_{1}=0.015$  $\lambda_{2}=0.005$  $\lambda_{3}=0.010$ | *25* | *Tamale* | *21.8 (0.7, 165.4)* | *95.9 (94.5, 97)* | *0.013 (0.005, 0.048)* | ***E*** | *464* |
| *3* | *Non-monotonic FOI* | $\lambda_{1}=0.014$  $\lambda_{2}=0.005$  $\lambda_{3}=0.009$ | *26* | *Kumasi* | *20.22 (0.9, 129.8)* | *96.5 (95.2, 97.5)* | *0.011 (0.006, 0.026)* | ***E*** | *495* |

*In the High FOI scenarios we assumed an FOI twice as high as the Cattarino et al FOI, while in the Low FOI scenarios we assumed half the Cattarino et al FOI. The median age of first infection was calculated as* $1 / (n\lambda)$ *where n is the number of circulating dengue serotypes, and in the time-varying scenario we estimated the average* $\lambda$ *as the population weighted average of* $\lambda_{1}, \lambda_{2}, \lambda_{3}$*using the Ghanian age-structure reported in the UNWPP data [21].*

- Line 260-266: we show the results obtained in the new analysis with higher and lower FOI as follows:

## *“The optimal testing scenario changes with the expected FOI*

*In a sensitivity analysis we tested the impact of assuming alternative FOI values for the three locations, as described in Table 1. Fig 4 summarises the results obtained under a high and low FOI, showing that in higher FOI settings testing younger age-groups is preferrable (scenario B), while in lower FOI settings testing of older age-groups (scenario E) is required to get accurate FOI estimates. In both cases the chosen scenario allowed to estimate the FOI with high precision and low uncertainty, as shown in Table 1.* *The model also fitted the simulated data, as shown in Fig S6.”*

- Line 276-293: we show the results obtained with models 2 and 3 as follows:

## *“The simulation framework works with decaying immunity and across transmission settings*

*The proposed simulation framework can successfully reconstruct the FOI also in scenarios with antibody decay and temporal changes in the intensity of transmission, including increasing, decreasing and non-monotonic transmission intensity patterns, and allows to identify an optimal testing scenario also when using these alternative assumptions on immunity decay and temporal changes in the FOI.*

*The selected testing scenarios varied across locations and with the modelled assumptions (Table 1). The simulation-based framework under model 2 could capture the seroprevalence data (Fig S7), and produced accurate estimates of the FOIs and decay rates (Fig S4), with a slightly larger uncertainty around the decay rate than in the FOI. Scenario A, which includes the minimum number of testes performed across all age-groups, was among the preferred scenarios for the antibody decay model (model 2). However, when assuming a high FOI, the chosen scenario was B, which involves the testing of the younger age-groups.*

*We found preferential testing of older age-groups (scenarios C and E) when using the time-varying FOI model (model 3), which can be explained by the fact that older age-groups are the only to provide information to estimate the earliest FOI (*$\lambda_{3}$*) in the time-varying model. The simulation framework can reconstruct the expected seroprevalence with good accuracy (Fig S8) and provides precise estimates of all FOIs across the three time-varying scenarios explored, with a slight increase in uncertainty for the earliest FOI (*$\lambda_{3}$*) (Fig S5).”*

- Line 315-321: we discuss the choice of model 1 for the case of Ghana as follows:

*“The proposed framework can determine the optimal testing scenario under different transmission intensity assumptions (constant FOI, time-varying FOI and with antibody decay) and estimate the FOI with high accuracy under the chosen scenario. Notably, different assumptions on the intensity of transmission as well as on the duration of immunity and on time-varying patterns of transmission influence the choice of the testing scenario. In the context of the SERODEN survey in Ghana, we used the simplest constant FOI model due to the evidence that dengue antibodies are long lasting across several transmission settings [10].”*

- We added Figures S3-S8 in the Supplementary information, to show the precision of our estimates under the chosen scenario selected with models 1, 2 and 3. All models can estimate the parameters of interest with high precision.

**3. Interpreting the differences between the 3 cities and lessons learned from different FOIs
The authors performed simulation-recovery experiments for the three cities; however, it does not seem like the parameter to be estimated (the FOI) differs much between them. Thus, we expected discussion as to why the chosen strategy differed for Kumsai and what lessons could be learned comparing the three cities. It would be more informative for readers to see how the optimal testing strategy differs for quite different FOIs and assumptions (e.g., very high and very low FOI; time-varying). For a constant FOI, it seems intuitive that preferencing testing in younger individuals would be optimal. There may be some neat results to be shown e.g., with age-of-first-infection. Does the optimal age-representation differ for a pathogen with a very young age-of-first-infection vs. later in life?**

This is a good point indeed, as extending the study to include higher and lower FOI values will help readers appreciate how preferential testing needs to be adjusted to the local transmission setting. In our original simulation study we used the FOI estimates predicted by Cattarino et al., and the chosen testing strategy differed between cities because this depends both on the FOI value assumed in the simulation study and on the available distribution of the samples (Figure 1 below), which differed between the cities.

Figure 1 Age-distribution of the samples available for Accra, Kumasi and Tamale under the baseline scenario 0.

We have now extended our simulation study to include higher and lower FOI values in model 1 (constant FOI) and we also explored how the choice of the testing scenario differs when testing alternative values for model 2 (i.e., a baseline scenario for the FOI and Ab decay, a high FOI and baseline Ab decay and a baseline FOI and high Ab decay value) as well as three different trends in the time-varying FOI (increasing, decreasing and non-monotonic). The key take home message is that higher FOI settings require testing younger age-groups, while in lower FOI settings it is necessary to give priority to testing older age-groups. To convey this message and to visually show this pattern we have included a new figure in the main text (Figure 4, also copied below). We also thank the reviewer for suggesting to include the median age-of-first-infection as an alternative measure to the average FOI, which can indeed be relevant from a clinical perspective.

**Summary of changes:**

- Table 1: we have summarised in Table 1 all models and parameterisations used, including the expected age of first infection as an alternative metric of the FOI.
- Line 260-266: we have presented the result from our analysis with higher and lower FOI values as follows:

## *“The optimal testing scenario changes with the expected FOI*

*In a sensitivity analysis we tested the impact of assuming alternative FOI values for the three locations, as described in Table 1. Fig 4 summarises the results obtained under a high and low FOI, showing that in higher FOI settings testing younger age-groups is preferrable (scenario B), while in lower FOI settings testing of older age-groups (scenario E) is required to get accurate FOI estimates. In both cases the chosen scenario allowed to estimate the FOI with high precision and low uncertainty, as shown in Table 1. The model also fitted the simulated data, as shown in Fig S6.”*

- We have included Figure 4, to shows how the chosen age-distribution of the samples changes based on the expected FOI. We added this figure to the main text as follows:

***Fig 4. Model fit for Accra under the 5-year age categorization and a high and low FOI.*** *The first row represents the seroprevalence using the chosen scenario, where the median and 95% credible interval of the estimated seroprevalence are reported in green as a line and shaded area, while the simulated dataset is reported in black, with the error bars indicating the exact binomial confidence interval. The second row illustrates the age-distributions of the tests with the chosen scenario (in green) for each city, compared to the available sample distribution in orange. The figure reports the results obtained with the high FOI (first column) and low FOI (second column).*

- In Figure S3 and S6 in the Supplementary Information we show respectively the goodness of fit and the accuracy of the FOI estimates, and how the choice of the testing strategy changes with the FOI.
- Line 322-328: we discuss the different choice of the optimal scenario for Kumasi compared to Accra and Tamale as follows:

*“Under this model, the testing scenarios selected in Ghana were scenario B for Accra and Tamale, and scenario D for Kumasi. Both scenarios require testing of the samples from young age-groups, which demonstrates the importance of recruiting children in seroprevalence studies. In the case of Kumasi, scenario B had a slightly higher bias and lower coverage compared to the baseline scenario, while scenario D not only decreased the bias but also increased the coverage while maintaining a low uncertainty around the estimate which made it the best one for this location. This demonstrates how the chosen testing scenario depends on both the transmission setting and the available number of samples.”*

**5. Figure 4 and iterative simulation-recovery framework
The iterative framework seems like a great idea, and it is interesting to think about how this 2-stage process might differ depending on our prior knowledge of the pathogen (e.g., entirely novel vs. new outbreak of well-understood pathogen). The authors could quite easily explore how this workflow would perform under different FOI assumptions using their simulation framework – simulate a representative serosurvey under various FOIs, and then perform the 2-stage uniform/tailored testing approach to find an optimal subset. This seems particularly pertinent given the authors rely on model-based FOI estimates as the ground truth for their dengue serosurvey – what if the true FOI is drastically different from the estimate from Cattarino et al, and the optimal sample distribution is different to the initial best-guess?**

This is another good point, and we are delighted to see that the reviewer agrees on the utility of the proposed two-step approach which helps indeed calibrate the study design in locations where prior information from the local setting is not available. As suggested, in the Supplementary Information we have added a whole section on the “Two-step testing approach” discussing how to distribute the second half of the samples to achieve the chosen distribution based on the analysis of the first half of the samples, and showing two examples where the FOI initially assumed was either too high or too low with respect to the (hypothetical) FOI obtained from testing half of the samples. We demonstrate that the accuracy of the estimates obtained with the two-step approach is not altered, even if the initial assumptions did not reflect the true transmission intensity.

**Summary of changes:**

- In the Supplementary material we added the following section:

# *“Two-step testing approach*

***Example distribution of the second half of the samples in the two-step testing process***

*In this section we show how it is possible to adjust the distribution of the samples if a too high or too low FOI was assumed in the initial phase of the study. Specifically, we show how to adjust the sample distribution in the hypothetical scenario where (i) the FOI estimate was half of the initially assumed value (this scenario was applied to the sample sizes collected in Accra) and (ii) the FOI estimate was twice the initially assumed value (this scenario was applied to the sample sizes collected in Kumasi).*

*Using the FOI estimate obtained from the interim analysis of half of the samples, we repeated the simulation study performed initially. In Fig S9, we compared the chosen scenarios selected based on the interim analysis (orange) and the initial study (green) for the two locations.*

***Figure S9:*** ***Comparison of sample distributions*** *obtained using the Cattarino et al. FOI estimates* *(“Old_scenario”, green) vs the sample distribution obtained if assuming half of the FOI value (left) and twice the FOI value (right) (“New_scenario”, orange).*

*It is hence possible to distribute the remaining samples available to match the newly chosen distribution (in orange in Fig S9) by (1) computing the total number of samples per age-group using the overall sample sizes available under the new optimal scenario (2) calculating the difference between the age-distribution in step (1) and the age-distribution of the samples already tested for the interim analysis (Fig S10).*

***Figure S10:*** ***Comparison of sample distributions*** *obtained using new FOI estimates (“New_scenario”, orange) vs the sample distribution of those tests performed in phase 1 under the old optimal scenario (“Tested”, green).*

*This difference represents the age-distribution needed for the second and final phase of the testing. If in an age-group more samples than those identified in the newly chosen scenario had already been tested in phase 1, we set the difference equal to zero (i.e., we do not add any more tests in that age-group). In the event that the samples sizes of the newly chosen scenario exceed the sample sizes of the old scenario, it is possible to rescale the new age-distribution by the number of samples available if the purchase of new tests is not possible. Fig S11 shows the final distribution achieved at the end of the study with the re-distribution method described in this section (Final_test) with the newly chosen scenario identified in the interim analysis (New_scenario).*

**Figure S11:** **Comparison between actual samples distribution** **achieved** (“Final_test”) **vs the optimal distribution** identified in the interim analysis (“New_scenario”).”

- Line 339-344: we discuss how our interim analysis is well placed to correct the sampling strategy based on FOI estimates obtained halfway through the analysis as follows:

*“In the Supporting Information we provide an example of how it is possible to adjust the distribution of the second half of the samples in a hypothetical situation where the FOI from the interim analysis (obtained from testing half of the samples in step 1) was different from the assumed one. We provided two examples exploring the case where the FOI estimate after step 1 was half than the assumed one, and another where the FOI was twice as big of the assumed value in the planning phase. The results are shown in the Supporting Information (Fig S9), and in both cases, our framework and the two-step approach allowed us to efficiently adapt the testing scheme to the new information and re-distribute the remaining samples accordingly to accurately estimate the FOI (Fig S9-S11).”*

**Minor comments
- Table 2 and Fig 1/Table 1 feel redundant together, suggest moving repeated information to the supplement and choosing one table or figure.**

We agree and have moved the two tables to the Supplementary Material (Table S1 and S11).

**- It might be useful to briefly explain how the sampling frame of the original SARS-CoV-2 study was chosen.**

In lines 95-96 we have added further details on the sampling strategy in the SARS-CoV-2 seroprevalence study, as follows:

*“The study was designed with a two-stage geographical cluster sampling method stratified by age and sex in urban households [20].”*

Further details van be found in the cited study.

**- We assume that the age distribution from the World Population Prospect 2021 was used for the ground-truth simulation, but we did not see that mentioned in the methods.**

We have cited the source in the caption of Figure 1 as follows:

*“****Fig 1. Blood sample distribution across several scenarios for the city of Accra using the 5-year age category.*** *Panel A shows the distribution of the available samples across the age groups and compares them with the population age structure of Ghana (World Population Prospect* [21]*). Panels B to F illustrate the sample distribution under scenarios A to E compared to the available samples in the baseline scenario 0 (which in the panel is indicated as “samples”).”*

**- Given Fig1A, would it be informative to add an extra comparison scenario where the age-distribution of the samples matches that of the population? Would that provide a better ground-truth than scenario 0?**

We don’t think that it would, because the premise of the work is leveraging on existing blood samples. Our sampling scheme can of course be applied also to the case where the sample distribution is representative of the population age-structure, but this would not add substantially to what is already presented in the paper. Notably, a key strength of the method is that it can be applied to the case where the samples are not representative, which often happens even with very accurate study designs especially in settings with young population age-structures due to difficulties in recruiting young children in these setting (and everywhere else).

**Reviewer #2: In this manuscript, Vicco and colleagues proposed a simulation-based method to assess the serosurvey designs using blood samples collected from previous surveys. Specifically, the authors applied their method to estimate dengue force of infection in Ghana, using samples previously collected for a SARS-CoV-2 serosurvey. The authors first simulated the age-dependent seroprevalence (Eq. 1) using the average yearly FOI per serotype that was estimated from Ref. [3], after which they used a binomial likelihood (Eq. 6) to reconstruct the posterior distribution of the force of infection. The authors tested several sampling scenarios to adjust the age distributions of samples.

The proposed statistical method looks reasonable, with a comprehensive simulation-based tests.**

Thank you for the positive feedback!

**I only have a few suggestions:

(1) Although using previous serosurvey provides a cost-effective data source for estimating dengue FOI, caution may be needed if two pathogens have some degree of cross-reactivity. For example, a few studies suggest the possible interaction between antibodies against SARS-CoV-2 and dengue virus [1]. The authors may briefly discuss measures to reduce this kind of bias:

Ref. [1]: Antibodies against the SARS-CoV-2 S1-RBD cross-react with dengue virus and hinder dengue pathogenesis, https://www.frontiersin.org/articles/10.3389/fimmu.2022.941923/full**

There does not seem to be a consensus on cross-reactivity between dengue and SARS-CoV-2, see for instance <https://pubmed.ncbi.nlm.nih.gov/35134813/>. Prof Malavige’s labs has further assessed this hypothesis on Sri Lankan samples without finding evidence of cross-reactivity either (unpublished work). We have taken steps to mitigate this risk in our study through testing a subset of the samples with a neutralisation assay (PRNT), which will indicate whether there are large discrepancies between the results obtained with the ELISA assay and the PRNT (taken as gold standard) through which we will adjust the population-level seroprevalence estimates using the likelihood-based method developed in Dorigatti et al. <https://www.nature.com/articles/s41467-021-24622-7>. We will further explore the opportunity to test this hypothesis on the Ghanian samples, given that we will know the individual-level serostatus to SARS-CoV-2 and dengue.

**(2) The authors assumed a uniform prior distribution between zero and one. Using weekly informative prior may be helpful to reduce the uncertainty.**

Thank you for the suggestion, we have verified that the use of more informative prior distribution (a normal distribution centred in 0.02 with standard deviation of 0.5) did not change the precision of the FOI estimates nor reduce the uncertainty. This is good news, because it clearly shows that there is information in the data to estimate the FOI even in the absence of prior information. We have included this result in Figure S2 of the Supplementary Material.

***“Figure S2:*** ***Comparison of FOI estimates with optimal scenario under two different prior distributions.*** *The plots show the FOI estimates obtained under the optimal scenario as median and 95% CrI represented with dot and error bar. It compares the Cattarino et al expected FOI [3] (orange) with the estimated one under model 1 respectively with a flat uniform prior distribution (blue) and informative normal prior distribution (green). The choice of the prior distribution does not influence the FOI estimate nor the credible interval.”*

**(3) The authors may wish to briefly discuss the reason for choosing a tolerance of 15%.**

Our rationale for selecting the 15% threshold was to ensure that the observed differences in bias with the baseline scenario remained within reasonable bounds. To ensure a robust and adaptable approach, we used a percentage of the upper bound of the bias to accept some tolerance and settled on 15% to strike a balance between precision and the available budget. We have included the following lines in the Discussion (lines 345-351):

*“Another limitation of the method we developed is the empirical choice of the tolerance used to determine sample sizes – such as the 15% difference from the* *upper bound of the 95% CrI of the baseline scenario – which can be tweaked depending on the targeted accuracy and available budget and resources. Nonetheless, the methodological framework developed in this study provides a rationale that can be applied to secondary testing of existing blood samples, including-in locations where there is limited previous information on the historical circulation and transmission intensity of a pathogen, while optimizing the accuracy of the FOI estimates and the use of resources.”*
