# Supplementary material for: A simulation-based method to inform serosurvey design for estimating the force of infection using existing blood samples
Source: PLoS Comput Biol. 2023 Nov 27;19(11):e1011666. doi: 10.1371/journal.pcbi.1011666 (PMC10727435; doi:10.1371/journal.pcbi.1011666)
Supplement: Attachment — Submitted filename: Point_by_point_response_Simstudy_Ghana_final.docx [file pcbi.1011666.s004.docx]

**Point-by-point response to reviewers**

**A simulation-based method to inform serosurvey designs for estimating the force of infection using existing blood samples**

Anna Vicco, Clare McCormack, Belen Pedrique, John H. Amuasi, Anthony Afum-Adjei Awuah, Christian Obirikorang, Nicole S. Struck, Eva Lorenz, Jürgen May, Isabela Ribeiro, Gathsaurie Neelika Malavige, Christl A. Donnelly, Ilaria Dorigatti

**Reviewer #1:**

**The authors have done a very thorough job of addressing our comments, and the paper is now suitable for publication. Great job!**

Thank you for the constructive feedback!

**One last comment: I appreciate the addition of the two-stage process simulation. However, it is not currently clear how the two-stage process actually helps with estimating FOI. The point of our suggestion was to demonstrate how the two-stage process actually improves the FOI estimates -- the new additions simply shows how the sample distribution is updated. I would suggest adding a figure with FOI on the y-axis, and on the x-axis: 1) the true FOI; 2) the FOI assumed for the initial study; 3) the FOI estimated under the “Old_scenario” and 4) the FOI estimated under the “New_scenario”. I believe the authors are trying to show that the FOI estimate under the new scenario is more accurate than under the old scenario, but that doesn’t currently come across.**

We thank the reviewer for this suggestion, we agree that this figure will help readers better understand the utility of adopting the two-step approach. As suggested, we have added a new figure within the *Two-step testing approach* paragraph in the Supplementary Information to visually illustrate the accuracy of the FOI estimate in the new scenario compared to the old scenario.

*Summary of changes:*

- Lines 173-183:

*“Fig S12 compares the FOI estimates obtained with the old scenario (selected from step I of the analysis) and the new scenario (selected from step II of the analysis) with the assumed FOI and true FOI values (respectively, half of the assumed FOI in Accra and twice of the assumed FOI in Kumasi). Fig. S12 shows that by changing the sample size distributions during phase II, it is possible to obtain unbiased FOI estimates that closely capture the true FOI estimate.*

***Figure S12:*** ***Comparison of FOI estimates obtained after phase I (“Old_scenario”) and after phase II (“New_scenario”) with the assumed and true FOI values.*** *Median (black point) and 95% CrI (black error bar) of the FOI estimates obtained after phase I ("Old_scenario”) and phase II (“New_scenario”) as compared to the assumed FOI (orange line) and true FOI (green line) in the hypothetical scenarios explored in this section.”*

**Minor points:
• L132: typo “reversible catalytic model” should be “models”
• L384, typo for “tests”
• Typo L389 "older age-groups are the only to"**

We thank the reviewer for pointing out the typos, we have now corrected them.

**The bias values in Table 1 are order of magnitude 1-20, but the definition is “absolute value of difference between the estimate FOI and that used to simulate the data”. Surely the bias should be of order 0.01? Is what is being shown the percentage difference?**

Indeed, we showed the percentage difference between the estimated FOI and the value used for simulating the data. To make this clearer, we have now included this detail in the methods section (line 165-169) and explicitly specified this also in Table 1 and in Figure 2.

*Summary of changes:*

- Lines 160-164: we have provided details on the expression of the bias and coverage in percentage terms as follows:
- ***Bias****: the absolute value of the difference between the estimated FOI* $\tilde{\lambda}$ *and the λ used to simulate the data divided by λ, expressed in percentage.*
- ***Coverage****: the number of times the FOI λ used to simulate the data falls within 95% CrI of the estimated FOI* $\tilde{\lambda}$ *across the 1,000 simulations, expressed in percentage.*
- Line 229-236: we have explicitly included in the caption of Fig 2 that the Bias and Coverage are expressed as percentages., as shown in tracked changes below:

- Line 249-252: We have explicitly added that the Bias and the Coverage are expressed as percentages in Table 1 as follows:

**It seems that with models 2 and 3, the bias and uncertainty both increase quite drastically – suggest discussing.**

Yes, the bias and uncertainty do increase with models 2 and 3. This is expected, given the number of estimated parameters is larger but the information provided is unchanged. We agree that it is useful to discuss these trends in the discussion.

*Summary of changes:*

Lines 324-327:

*“Moreover, we found that the FOI estimates obtained with model 2 (with antibody decay) and model 3 (with time-varying FOI) exhibit larger bias and uncertainty compared to the estimates obtained with model 1, which is expected given the larger number of parameters estimated using the same data.”*

**Figure 3 is presumably for model 1 – please specify.**

Yes, Figure 3 pertains to model 1. We have now explicitly specified this in the text for clarity. Furthermore, we have harmonized the colour scheme in Figure 3 with the other figures and the rest of the manuscript, where green has been used to represent the optimal scenario.

*Summary of changes:*

- Line 257-264:

***Fig 3. Model fit for the three cities under the 5-year age categorization with model 1.*** *The first row represents the fit for scenario 0, where the median and 95% CrI of the estimated seroprevalence are reported in orange as a line and shaded area, respectively. The second row illustrates the model fit with the selected scenario in green. In both panels, the datasets used for model fitting are shown in black, with the error bars showing the exact binomial 95% Confidence Interval. The third row shows a comparison of the estimated seroprevalence in scenario 0 (orange) and the optimal scenario (green).*
